# Bioprospecting of culturable marine biofilm bacteria for novel antimicrobial peptides

**DOI:** 10.1002/imt2.244

**Published:** 2024-10-17

**Authors:** Shen Fan, Peng Qin, Jie Lu, Shuaitao Wang, Jie Zhang, Yan Wang, Aifang Cheng, Yan Cao, Wei Ding, Weipeng Zhang

**Affiliations:** ^1^ MOE Key Laboratory of Evolution & Marine Biodiversity and Institute of Evolution & Marine Biodiversity Ocean University of China Qingdao China; ^2^ Department of Biomedical Sciences, Faculty of Health Sciences University of Macau Taipa Macao SAR China; ^3^ College of Pulmonary & Critical Care Medicine Chinese PLA General Hospital Beijing China; ^4^ MOE Key Laboratory of Marine Genetics & Breeding and College of Marine Life Sciences Ocean University of China Qingdao China

**Keywords:** antimicrobial peptide, deep learning, marine biofilm, marine resource, Ribo‐seq

## Abstract

Antimicrobial peptides (AMPs) have become a viable source of novel antibiotics that are effective against human pathogenic bacteria. In this study, we construct a bank of culturable marine biofilm bacteria constituting 713 strains and their nearly complete genomes and predict AMPs using ribosome profiling and deep learning. Compared with previous approaches, ribosome profiling has improved the identification and validation of small open reading frames (sORFs) for AMP prediction. Among the 80,430 expressed sORFs, 341 are identified as candidate AMPs with high probability. Most potential AMPs have less than 40% similarity in their amino acid sequence compared to those listed in public databases. Furthermore, these AMPs are associated with bacterial groups that are not previously known to produce AMPs. Therefore, our deep learning model has acquired characteristics of unfamiliar AMPs. Chemical synthesis of 60 potential AMP sequences yields 54 compounds with antimicrobial activity, including potent inhibitory effects on various drug‐resistant human pathogens. This study extends the range of AMP compounds by investigating marine biofilm microbiomes using a novel approach, accelerating AMP discovery.

## Highlights

- Bacterial isolation, cultivation, and genome sequencing have laid the foundation for antimicrobial peptide (AMP) discovery from marine biofilms, where AMPs are largely unexplored.
- The incorporation of ribosome profiling has enhanced the sequence filtering process, enabling more accurate identification of small open reading frames before AMP prediction.
- A multilayered model‐based filtering pipeline exhibits outstanding predictive performance in screening candidate AMPs.
- Experimentally validated AMPs show excellent antibacterial efficacy, highlighting their potential as candidate drugs.

## Graphical abstract

In the context of marine resource bioprospecting, we construct a bank of culturable marine biofilm bacteria encompassing 713 strains and their nearly complete genomes. Using ribosome profiling and a new deep learning algorithm, we get more than 300 candidate antimicrobial peptides (AMPs). We further conduct antimicrobial activity and cytotoxicity experiments to evaluate their potential as druggable molecules.

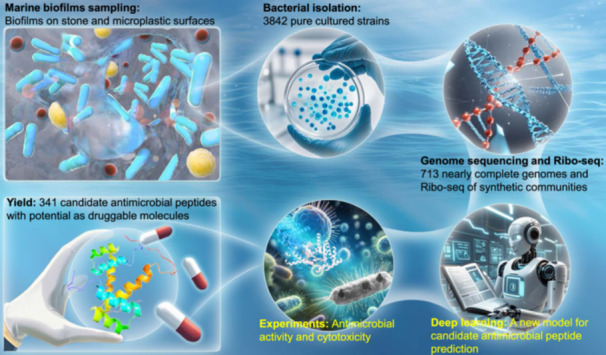

## INTRODUCTION

Antimicrobial resistance has emerged as a significant public health threat, suggesting we fall short of effective antibiotics [1, 2]. Hence, searching for novel antimicrobial drugs could serve as an attempt to instigate a competitive struggle against bacteria. Antimicrobial peptides (AMPs) have attracted significant attention as possible therapeutic agents in the search for novel antibiotics. AMPs encompass diverse bioactive compounds that animals, plants, and microbes can produce to combat bacterial infections [3]. AMPs are amphiphilic small molecules with broad‐spectrum antibacterial effects employing mechanisms like destroying bacterial cell membranes and interfering with DNA or RNA synthesis [4, 5].

AMPs can be synthesized through the nonribosomal pathway or the ribosomal pathway. Among these, many AMPs synthesized by ribosomes exhibit potent antibacterial activity without modifications [6, 7]. The search for AMPs is limited by their unique characteristics, represented by short sequences and low identities [8]. Artificial intelligence enables the identification of sequence aspects that are not perceptible to humans. In particular, applying deep learning techniques to extract AMPs from massive peptide sequences has significantly improved the identification of novel AMPs [9, 10, 11, 12]. For instance, diverse candidate AMPs from human gut microbiomes have been systematically explored through multiple deep‐learning models [11]. These peptide sequences were further filtered through metaproteomics, which resulted in 2349 candidate AMPs [11]. However, there is ample opportunity for improvement in the pipelines used for AMP prediction. In particular, it is difficult to identify small open reading frames (sORFs) for AMP prediction.

Moreover, AMPs of bacterial origin represent only 12% of the sequences in the well‐known APD3 database [13], with nearly 40% of AMPs in clinical trials being of human origin [14]. Any evolved resistance to these AMPs may lead to collateral resistance to human immunity [14]. As a result, AMP mining should encompass a broader range of lifestyles and environments. In particular, the diversity and function of AMPs produced by marine bacteria are yet to be thoroughly investigated. Due to the high salinity, low temperature, and restricted nutrients in aquatic habitats, bacteria living in such environments frequently produce metabolites with unique structures and functions [15]. In addition, marine bacteria play a crucial role as repositories of natural products and potential drugs, such as which are effective against fungi and drug‐resistant pathogenic bacteria [16, 17].

In marine environments, biofilms are microbial communities adhering to any immersed substrates, such as artificial substrates [18], stone (ST) surfaces [19, 20, 21], microplastics (MPs) [22], or animal guts [23, 24]. A global survey of marine biofilms has shown much higher species diversity than planktonic microorganisms, with as much as 60% of the genes in biofilm‐associated bacteria being functionally unknown [25, 26, 27]. In particular, biofilms on coastal ST surfaces harbor microbes with rich biosynthetic potential [21], and biofilms on MPs were attached by microbes with strong motility and antibiotic resistance [22]. In addition, more signal transduction pathways within biofilms suggest that interactions between biofilm‐associated bacteria are more robust than between free‐living bacteria [25, 26]. Thus, biofilm‐associated bacteria are likely to produce metabolites as a defense during the competition for territory, suggesting a greater potential for discovering novel AMPs.

The lack of relevant omics resources (genomic, transcriptomic, and proteomic data) and suitable processes limit the extraction of AMPs from marine biofilms. In the present study, we performed a large‐scale isolation of bacterial strains from marine biofilms and then sequenced their genomes. Following that, to find potential AMPs from the genomes, we used a novel method that combined ribosome profiling (Ribo‐seq) with an enhanced deep‐learning model. We highlight the benefits of employing Ribo‐seq to identify and isolate expressed sORFs from intricate communities containing numerous diverse strains. The deep‐learning model consists of a convolutional neural network (CNN), two bidirectional long short‐term memory (Bi‐LSTM) layers, and an Attention layer. This model employs a “coarse‐to‐fine” learning approach to predict AMP sequences efficiently. In attempts to achieve this objective, potential AMPs with strong antibacterial properties and minimal cytotoxicity were acquired.

## RESULTS

### Bacterial isolates and genome information

**Figure 1:**
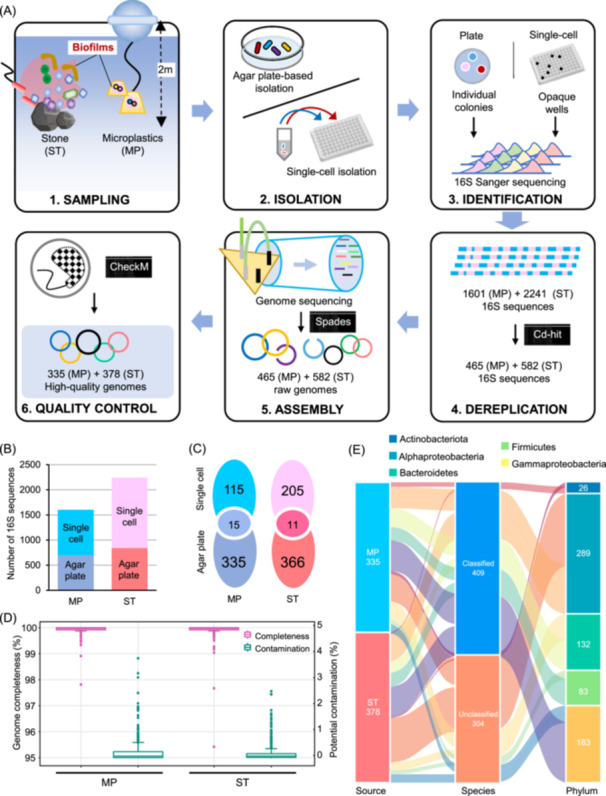
Collection and genome sequencing of bacterial strains from marine biofilms. (A) The workflow for collecting and archiving bacterial strains from marine biofilms using two approaches. Biofilms were sourced from microplastics (MP) and stones (ST) to isolate strains using the agar plate‐based and single‐cell isolation methods. The isolates were then identified based on 16S rRNA gene Sanger sequencing. Strains with nonredundant 16S rRNA sequences were selected for whole genome sequencing, and the resulting assembled genomes were subjected to quality control to generate 713 high‐quality genomes. (B) The number of 16S rRNA genes generated during bacterial isolation using agar plate‐based and single‐cell isolation from biofilms on MP and ST. (C) Venn analysis of nonredundant 16S rRNA sequences generated from the two isolation approaches. (D) Completeness and potential contamination of the genomes. In a boxplot, the central line represents the median, bounds represent the upper and lower quartiles, whiskers represent the maximum and minimum. (E) Phylum‐level composition and percentage of genomes that could not be classified into the species level based on the Genome Taxonomy Database.

Bacterial strains were obtained from two distinct forms of coastal marine biofilms, specifically biofilms found on MP and those on ST surfaces. While diluting and spreading environmental microbiota samples on agar plates for bacterial isolation, it was frequently observed that colonies established by specific strains showed delayed growth and were later covered by other strains (Figure S1). This phenomenon motivated us to culture the strains separately by using two different approaches. After collecting MP and ST biofilm samples, bacterial strains were isolated using the traditional agar‐plate streaking method by following the dilution‐to‐extinction approach (Figure 1A). In parallel, samples were subjected to strain isolation using a single‐cell method based on the limiting dilution strategy (Figure 1A). Scanning electron microscope (SEM) observation was conducted on selected strains to show that the isolates are probably pure (Figure S2). In total, 3842 16S rRNA gene sequences were obtained, including 700 sequences of MP strains derived from the agar plate‐based isolation (MP_Plate), 901 sequences of MP strains from the single‐cell isolation (MP_Single_cell), 843 sequences of ST strains from the agar plate‐based isolation (ST_Plate), and 1398 sequences of ST strains from the single‐cell isolation (ST_Single_cell) (Figure 1B). After classifying the amplicon sequence variants (ASVs), 465 ASVs were obtained for the MP biofilms, with only 15 of these shared between the MP_Plate and MP_Single_cell. Similarly, 582 ASVs were obtained for ST biofilms, of which only 11 were common to the two methods (Figure 1C). Furthermore, we conducted rarefaction analysis on the 16S sequences and operational taxonomic units (OTUs) clustered at a 97% similarity level. This study allowed us to determine that the single‐cell culturing method has significantly enhanced the results obtained from agar‐plate culture (Figure S3). Simultaneously, most species still required culture (Figure S3).

The 465 MP‐derived and 582 ST‐derived strains, deemed nonredundant based on similarity analysis of 16S rRNA gene sequences, were subjected to genome sequencing. After quality control, 335 and 378 high‐quality (completeness >95% and contamination <5%) genomes were obtained for MP and ST, respectively, with the genome information in Table S1. Importantly, there was a complete absence of similarity between the bacterial genomes extracted from MP and those from the ST biofilms. Over 50% of the genomes in both environments were over 99.5% complete, and their contamination levels were below 0.5% (Figure 1D). Regarding the genome size, the MP bacteria ranged from 2.5 to 6.4 Mb, while the ST bacteria ranged from 2.5 to 6.5 Mb (Figure S4A). The number of contigs ranged from 1 to 205, while for the ST bacteria, the number ranged from 1 to 167 (Figure S4B). The maximum contig length reached 4.4 and 5.1 Mb for MP and ST genomes, respectively (Figure S4C), while their average contig N50 was 1.0 and 1.5 Mb, respectively (Figure S4D). The genomes were merged and analyzed using average nucleotide identity (ANI) analysis. The findings indicated that all the pairs had ANI values lower than 99.9% (Figure S5A), suggesting that they were distinct strains. In particular, out of the 253,828 genome pairings, 237,106 pairs (93.4%) had an ANI below 76, indicating significant genetic differences (Figure S5B).

Taxonomic annotation was then performed on the genomes using the Genome Taxonomy Database (GTDB). At the phylum level (class level for Proteobacteria), the strains were assigned to Alphaproteobacteria, Gammaproteobacteria, Bacteroidetes, Firmicutes, and Actinobacteria (Figure 1E). According to the GTDB annotation, MP and ST possessed 138 and 166 genomes (304, accounting for 42.6% of all strains) that could not be classified to the species level (Figure 1E). The isolated strains were distributed across 44 families at the family level, many of which were endemic to marine environments, such as Alteromonadaceae, Rhodobacteraceae, and Flavobacteriaceae (Figure S6). At the genus level, *Qipengyuania*, *Psychrobacter*, and *Pseudoalteromonas* were overrepresented taxa (Figure S7). The Venn analysis comparing bacteria originating from MP and ST indicated that many distinct taxa were also identified while shared families and genera existed between these two environments (Figure S8). Moreover, open reading frames (ORFs) were predicted from the 713 genomes to reveal the functional novelty of the isolated bacteria. These ORFs were then annotated using the Kyoto Encyclopedia of Genes and Genomes (KEGGs). For the MP and ST strains, the median ORFs per genome values were 3320 and 3429, respectively (Figure S9A). The median values of the annotated ORFs per genome were 1766 and 1891 (Figure S9B), and the median ratios of annotation were 54.4% and 55.7% (Figure S9C) for the MP and ST strains, respectively.

### A pipeline integrating Ribo‐seq and deep learning

**Figure 2:**
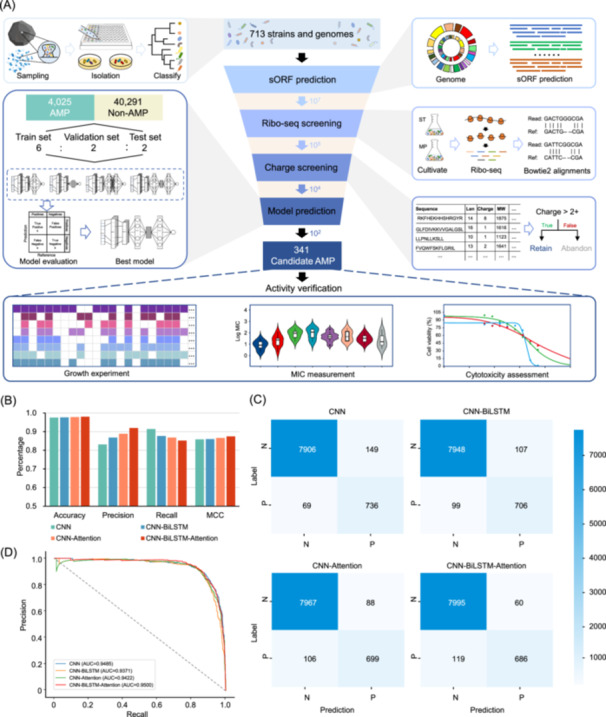
Workflow of antimicrobial peptide (AMP) discovery and deep learning model evaluation. (A) The whole workflow for the discovery of candidate AMPs from marine biofilms. Nonredundant bacterial strains were isolated from marine biofilms, and their genomes were sequenced. Small open reading frames (sORFs) were identified from the genomes by combining genomics and Ribo‐seq. The identified sORFs were evaluated by a newly developed deep‐learning model. Activity and cell toxicity of 60 predicted AMPs were verified through experiments. (B) Accuracy, precision, recall, and Matthews Correlation Coefficient (MCC) of the four models. (C) Confusion matrices to visualize the correlations between the labeled and the predicted AMPs. “N” and “P” represent the negative and positive samples. (D) Area Under the Precision‐Recall Curve (AUPRC) results. The CNN‐BiLSTM‐Attention model achieved the highest score (0.9500).

The pipeline of AMP prediction from the 713 genomes of marine biofilm strains is shown in Figure 2A. We employed neural network algorithms to develop four models trained using 4025 AMP sequences documented in public databases (APD3, DBAASP, dbAMP, and DRAMP) (Figure S10). Many of these AMPs were discovered in animals (Figure S10). Firmicutes and Actinobacteria were the primary sources of those found in bacteria (Figure S10). The detailed steps of data partitioning of the previously documented AMPs are described in the method section. A one‐dimensional CNN model was trained and employed as the basis for the training of other models (Figure S11A). Attention layers were introduced to capture key sequence features and information (Figure S11B). To evaluate the potential interactions between amino acids at different positions within a peptide, the initial model was enhanced by incorporating two BiLSTM layers. These layers simultaneously consider the peptide's temporal dependency and sequence information in the overall context (Figure S11C). Finally, a hybrid model incorporating the CNN, BiLSTM, and Attention layers was established to enhance computational efficiency and prediction accuracy further (Figure S11D). Model testing revealed that the hybrid CNN‐BiLSTM‐Attention model had the highest precision (92%), with a notable improvement of 3%–9% compared to other models (Figure 2B). This model also showed exceptionally high accuracy (98%) and Matthews Correlation Coefficient (MCC, 87%) (Figure 2B). The confusion matrices for the four models revealed that the hybrid model could attain a harmonious balance between precision and recall (Figure 2C). Moreover, the ranking of the Area Under the Precision‐Recall Curve (AUPRC) further confirmed the better performance of the CNN‐BiLSTM‐Attention model regarding comprehensive performance (Figure 2D). Furthermore, we assessed the efficacy of our CNN‐BiLSTM‐Attention model compared to other pre‐existing models by employing the identical test data set. The findings demonstrate that our model outperforms other models in terms of accuracy, precision, and MCC (Figure S12). Hence, the CNN‐BiLSTM‐Attention model was selected to predict AMPs in the following steps.

EMBOSS was used to predict sORFs before candidate AMP prediction, producing 88,358,942 sequences. In total, 45,384,259 of these sORFs were from MP strains, and 42,974,683 came from ST strains. For MP and ST biofilms, dereplication revealed 33,491,575 and 33,985,363 nonredundant sORFs, respectively. Since accurately predicting sORFs and identifying which sORFs are expressed is difficult, we employed Ribo‐seq. Two communities were established by pooling strains from the same biofilm niche to create a synthetic community for sample preparation. Following an overnight culture period, the two communities produced 25.20 and 27.32 Gb data sets for the MP and ST biofilms, respectively (Table S2). The reads from Ribo‐seq were mapped to the bacterial genomes to investigate the presence of all strains in the synthesized communities (Figure S13). All the genomes were matched, each recruiting more than 200 reads (Figure S13), suggesting that all the strains are present in the synthesized communities. Ribo‐seq reads were then mapped to the nonredundant sORFs generated in the previous step, yielding 78,454 and 72,542 sORFs in the MP and ST biofilms, respectively. Then, all the expressed sORFs from the two communities were pooled to yield 150,996 nonredundant sequences. AMPs typically exhibit amphiphilic cationic characteristics [14, 27, 28]. Hence, the net charges of the sequences were calculated to finally yield 80,430 sequences with a net charge >2 for candidate AMP prediction. The 80,430 sequences were distributed across 44 bacterial families, among which family Alteromonadaceae was the most prevalent (accounting for 23%), followed by Rhodobacteraceae (21%), Flavobacteriaceae (15%), and Sphingomonadaceae (10%) (Figure S14).

### Sequence characteristics of the candidate AMPs

**Figure 3:**
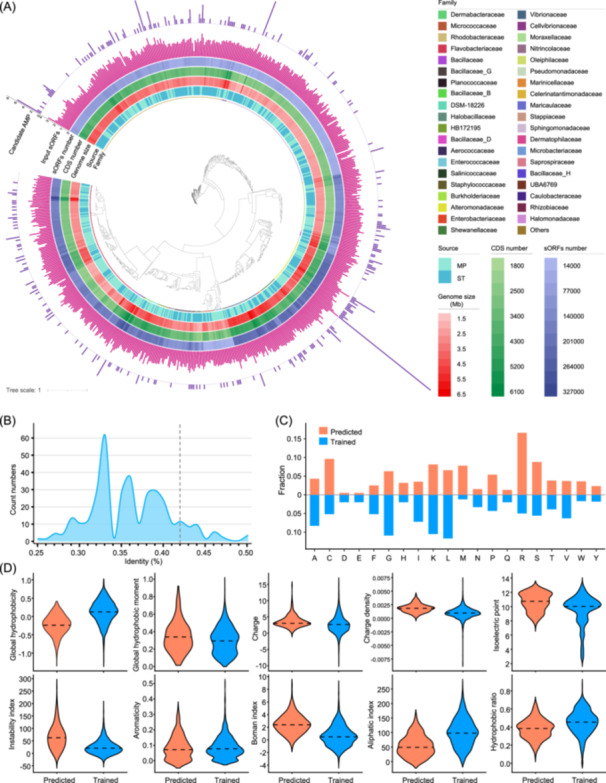
Overall distribution profile of predicted candidate AMPs across bacterial phylogeny and their sequence characteristics. (A) Candidate AMPs (Cand‐number) and sORFs were distributed across families of the 713 nonredundant bacterial strains isolated from marine biofilms. The tree was constructed based on whole genomes using the maximum likelihood method. The outermost ring represents the distribution of candidate AMPs (*n* = 341) as predicted by the deep‐learning model. The second ring represents the distribution of sORFs (*n* = 80,430) used as input for the deep learning model. Information on the sORFs number, the number of coding sequences (CDS), genome size, bacterial source, and Family‐level affiliation are also displayed. (B) Identities between the predicted AMPs and those in the training data set. (C) Comparative view of the amino acid frequency in the predicted AMPs and those in the training data set. (D) A comparison of sequence properties, including global hydrophobicity and hydrophobic moment, charge, charge density, isoelectric point, instability index, aromaticity, Boman index, aliphatic index, and hydrophobic ratio of the predicted AMPs and those in the training data set. The long‐dashed line represents the median.

Candidate AMPs were sequentially predicted through the deep learning model, identifying 341 sequences of the 80,430 sORFs as candidate AMPs. The sequence list of these candidate AMPs is shown in Table S3. Distribution patterns of the candidate AMPs, input sORFs (expressed and positively charged sORFs used as the query of AMP prediction), and coding sequence (CDS) across the bacterial phylogenetic tree are shown in Figure 3A. While candidate AMPs were distributed across diverse branches, their enrichment, particularly taxa, could be observed (Figure 3A). Furthermore, the potential AMPs were dispersed throughout 33 different bacterial families, with Rhodobacteraceae, Alteromonadaceae, and Flavobacteriaceae being the most prevalent (Figure S15). Interestingly, many families found here, including Halomonadaceae, Salinicoccaceae, and a few unnamed families (i.e., DSM‐18226 and HB172195), had no previously recorded AMPs. Regarding sequence novelty, 85% of these candidate AMPs exhibited <40% identity to known AMP sequences in the training data set, with a maximum of 50% identity (Figure 3B). The Met, Arg, Trp, Cys, His, and Ser residues in the candidate AMPs were 1.58 to 6.47 times more common than those in the training data set (Figure 3C). The physical and chemical properties of these AMPs, such as global hydrophobicity, were also significantly dissimilar to those noted in the training data set (Figure 3D). For example, lower global hydrophobicity and hydrophobic ratios were found for the marine biofilm candidate AMPs compared with those from the training data set (Figure 3D). In addition, AMPs derived from MP and ST have consistent properties in terms of sequence identity (Figure S16), amino acid frequency (Figure S17), and sequence physicochemical characteristics (Figure S18), indicating that the AMP characteristic may not be affected by the biofilm niche. These findings showed that our model can accurately identify AMPs and has acquired latent sequence properties. The low identity and particular taxonomic relationship with previously identified AMPs suggested the presence of highly unique AMPs from marine biofilm bacteria.

### Antimicrobial effect and cytotoxicity

**Figure 4:**
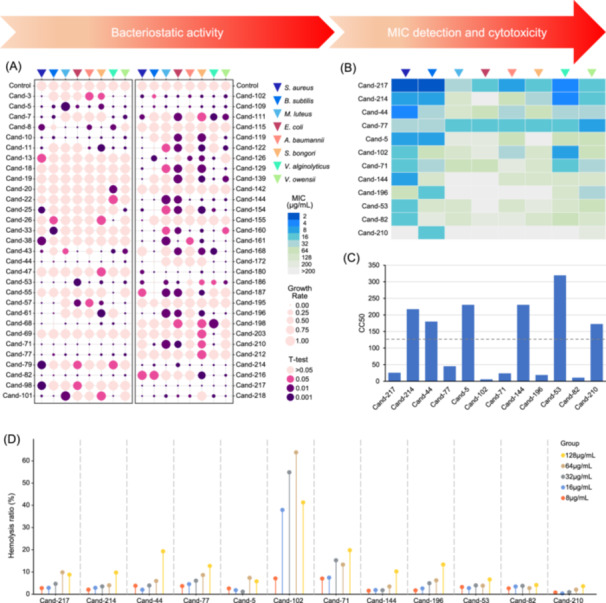
Antibacterial effect and cytotoxicity of selected candidate AMP (Cand‐number) molecules. (A) Antibacterial effect of 60 AMPs against eight pathogenic bacterial strains. Full names: *Staphylococcus aureus* ATCC 12600, *Bacillus subtilis* ATCC 23857, *Micrococcus luteus* ATCC 4698, *Escherichia coli* ATCC 11775, *Acinetobacter baumannii* ATCC 19606, *Salmonella bongori* ATCC43975, *Vibrio alginolyticus* xv22, and *Vibrio owensii* ems001. The inhibitory effect of AMPs was evaluated after comparing the cell density of bacteria grown with AMP molecules (200 μg/mL) to the control group (bacteria grown without AMP molecules). The bubble size indicates cell density, and the bubble color indicates the *p*‐value generated by the Students' *t*‐test. (B) Minimum inhibitory concentration (MIC) of 12 selected candidate AMP molecules. (C) Cytotoxicity of 12 selected candidate AMPs against human normal colon epithelial cells (NCM‐460). The assay was conducted using the 3‐[4,5‐dimethylthiazol‐2‐yl]−2,5 diphenyl tetrazolium bromide method and CC50 values are shown. (D) Hemolysis of sheep red blood cells by different concentrations of AMPs. All experiments were conducted with three independent biological replicates.

The functions of the candidate AMPs were validated after randomly selecting 60 from the top 100 sequences (in terms of probability) for chemical synthesis. Antibacterial effects of these AMPs were examined against eight pathogenic bacterial strains, including three Gram‐positive bacteria: *Staphylococcus aureus* ATCC 12600, *Bacillus subtilis* ATCC 23857, and *Micrococcus luteus* ATCC 4698, and five Gram‐negative bacteria: *Escherichia coli* ATCC 11775, *Acinetobacter baumannii* ATCC 19606, *Salmonella bongori* ATCC 43975, *Vibrio alginolyticus* xv22, and *Vibrio owensii* ems001. Several of these strains are marked as multi‐drug‐resistant strains (Table S4). Among the 60 examined candidate AMPs, 54 exhibited antibacterial activity against at least one bacterium, yielding an effectiveness rate of 90% (Figure 4A). We then selected the 12 candidate AMPs with strong broad‐spectrum antibacterial effects to measure minimum inhibitory concentration (MIC). Each AMP exhibited robust inhibitory activity (MIC ≤ 32 μg/mL) [12] against at least one bacterium (Figure 4B). Eight of these AMPs displayed a high antibacterial effect (MIC ≤ 8 μg/mL) against at least one of the above‐mentioned bacterial pathogens (Figure 4B). Two AMPs (Cand‐217 and Cand‐77) were highly efficient and exhibited broad‐spectrum antibacterial activity against nearly all strains, with MIC values ≤32 μg/mL (Figure 4B). In particular, the MIC of Cand‐217 against the multi‐drug‐resistant bacterium *S. aureus* was 2 μg/mL (Figure 4B). Moreover, the cytotoxicity of 12 candidates, as mentioned above, on human normal colon epithelial cells (NCM‐460) was assessed (Figure 4C). Cytotoxicity assays were conducted with the candidate AMPs at various concentrations, and the respective CC50 values were estimated. Six (Cand‐214, Cand‐44, Cand‐5, Cand‐144, Cand‐53, and Cand‐210) of the 12 AMPs exhibited low cytotoxicity (CC50 > 128 μg/mL) (Figure 4C), suggesting their good potential as drug candidates. Additionally, the hemolysis of sheep red blood cells by various AMP concentrations was investigated (Figure 4D). Of the 12 AMPs examined, only two (Cand‐102 and Cand‐71) showed over 10% hemolysis at a concentration of 64 μg/mL (Figure 4D). Of the 12 AMPs examined, six (Cand‐217, Cand‐214, Cand‐5, Cand‐53, Cand‐82, and Cand‐210) showed less than 10% hemolysis at a concentration of 128 μg/mL (Figure 4D). These findings revealed minimal toxicity to the animal cells.

### Antimicrobial mechanisms

**Figure 5:**
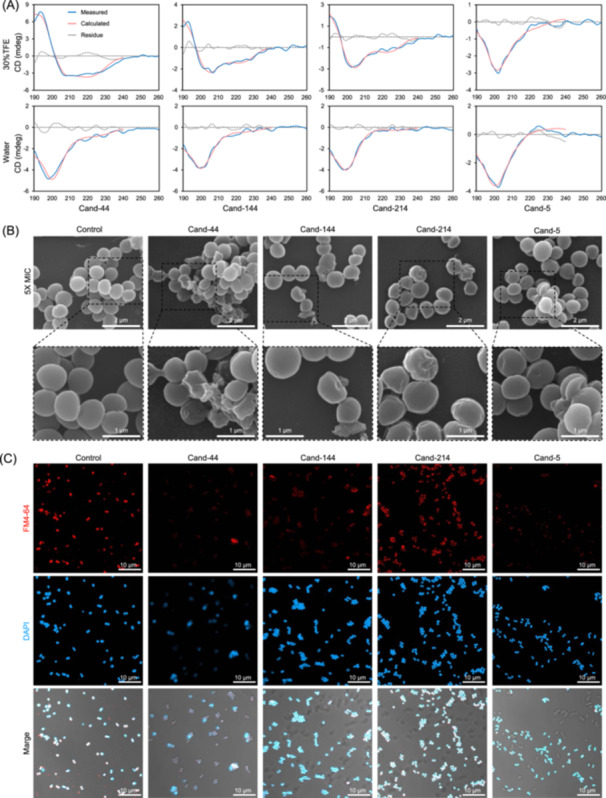
Circular dichroism (CD) spectrometer study of four AMP molecules (Cand‐number) and microscopy observation of their effect on *S. aureus* ATCC 12600. (A) CD spectra of four AMP molecules dissolved in 30% trifluoroethanol (TFE) solution (upper panel) and water (lower panel). (B) Scanning electron microscope (SEM) images of *S. aureus* ATCC 12600 without (control) and with treatment of AMP molecules. (C) Fluorescence microscopy images of *S. aureus* ATCC 12600 without (control) and with treatment of AMP molecules. Bacterial cells were stained with FM4‐64 and 4′,6‐diamidino‐2‐phenylindole (DAPI). The merged image includes the FM4‐64 field, the DAPI field, and a bright field. Dose of the AMP molecules: 20 μM for all the AMPs in the CD spectrometer study, 5 × MIC in the SEM observation, and 1 × MIC in the fluorescence microscopy observation.

To explore the mechanisms of the candidate AMPs, the structures of four AMPs (Cand‐44, Cand‐144, Cand‐214, and Cand‐5) with potent antimicrobial activity were predicted using AlphaFold2. Regular α‐helix structures were predicted for Cand‐44, Cand‐144, and Cand‐214, while partial α‐helix structure was detected for Cand‐5, which also contained random curls (Figure S19). The α‐helix structures of the four AMPs were further supported by the predicted helical wheel representations of amino acid sequences (Figure S20). In particular, the helix of Cand‐44 had one side with mainly polar residues and the other with primarily hydrophobic residues (Figure S20). Subsequently, the secondary structures of the four peptides were investigated through a circular dichroism (CD) spectrometer (Figure 5A). In the 30% trifluoroethanol (TFE) solution mimicking hydrophobic conditions of the cell membrane, Cand‐44, Cand‐144, and Cand‐214 displayed typical α‐helix structures (Figure 5A), consistent with the results of AlphaFold2. Based on previous understanding of AMPs, they may target the bacterial cell membranes [29]. SEM was used to examine *S. aureus* ATCC 12600 cell morphologies with and without the four AMPs (5 × MIC) treatment to test this hypothesis (Figure 5B). As demonstrated by the electron microscopy images, the treated cells' membrane structure changed, with some fading (Figure 5B). Moreover, a confocal laser scanning microscope (CLSM) was used to observe *S. aureus* ATCC 12600 that was exposed to the four AMPs (1 × MIC) (Figure 5C). The bacterial cells were stained with the membrane dye FM4‐64 and the nucleic acid stain DAPI. As a result, AMPs did not affect the nucleic acids stained with DAPI, although FM4‐64 signals were significantly weaker (Figure 5C). These findings indicated that the four AMPs investigated are likely to target the cell membrane of *S. aureus* ATCC 12600.

## DISCUSSION

This study is based on large‐scale bacterial isolation and genome sequencing and represents the first systematic mining of candidate AMPs from marine biofilms. We also present a new pipeline combining ribosome profiling and deep learning to identify candidate AMPs.

Although genomic information of marine biofilm‐associated microbes has been revealed by metagenomics, these genomes are incomplete [30]. Moreover, the absence of culturable strains impedes the further generation of paired information, such as the proteome and translatome. These challenges motivated us to undertake bacterial isolation from marine biofilms. The relatively small overlaps between ASVs derived from the two biofilm niches, namely MP and ST, suggest that the physical and chemical properties of MP and ST surfaces may have selected distinct species during the assembly of the biofilm communities. Certain species, which are rare in in situ marine environments, can be isolated and cultivated in laboratory conditions. Following this scenario, it is speculated that many novel species in marine biofilm niches remain uncultivated. Our results also suggest that using two complementary isolation approaches (i.e., plate streaking and single‐cell isolation) has enhanced the cultivation of nonredundant strains. The bacterial growing state may be the reason for the modest yield overlap between the two approaches. An essential phenotype distinguishing marine biofilm‐derived bacteria and facilitating their isolation is likely the ability to grow as colony‐forming or planktonic. The bacterial strain and genome resources in this study have established the groundwork for extracting AMPs from marine biofilms.

Prediction of sORFs from genomes is challenging, as it may generate sORFs with considerable false discovery. Although proteomics can, in theory, detect small peptides, it usually has low measurement depth, capturing only about 10%–20% of peptides [31]. Moreover, proteomic spectra tend to be saturated with abundant proteins from dominant microbial species or conserved gene families, and this issue is improbable to be addressed by increasing the speed or time of mass spectrum scan [32]. The above‐mentioned issues are especially apparent in complicated microbial communities, such as marine biofilms, resulting in significant data loss. On the other hand, Ribo‐seq can directly detect RNA fragments shielded by ribosomes and provide a temporal view of peptide sequences that are now being translated [33, 34, 35]. Ribo‐seq provides high‐throughput data sets as it is performed on the Illumina platform. Accordingly, we provide evidence that Ribo‐seq could recover 0.1709% small peptides from all the predicted possible sORFs, with a higher recovery rate than the previously used proteomics approach [11].

Following the utilization of Ribo‐seq, we develop a deep‐learning model for AMP prediction. Even though deep learning is widely applied across many domains, there is always potential for advancement. We constructed several sequence feature recognition models and identified the best one here, considering various architectures' possible attention bias and generalization capacities. The model provides an effective pipeline for AMP prediction by integrating CNN with two BiLSTM layers, an Attention layer. Individual CNN models perform poorly when it comes to prediction accuracy. Increasing the number of sequence feature collecting layers enhances the model's prediction performance. Further, when multiple layers are utilized together, the precision reaches up to 92%. Notably, most of the 341 candidate AMP sequences obtained in the final prediction exhibited <40% identity to the training data, with significantly higher numbers of Trp and Arg, which were thought to be associated with antibacterial properties [36]. These characteristics demonstrate that our model depends not only on amino acid signatures or sequence similarity. There are few sequences from marine habitats in the data sets used to train the algorithm, supporting its ability to identify unknown sequences accurately. Our findings show that a single multi‐layer model may accurately and successfully identify putative AMPs. This tool is suitable for analyzing data sets containing microorganisms present in natural marine habitats. Furthermore, similar sequence characteristics between AMPs from MP and ST suggest that the biofilm niche type (e.g., substrate type, including biofilms on different MP materials) has little influence on the mining of AMPs.

Following the bioinformatic prediction, the synthesized candidate AMP molecules exhibit robust antibacterial effects against Gram‐positive and Gram‐negative bacteria. Among them, *S. aureus* is one of the top four foodborne pathogens, according to the World Health Organization [37]. Interestingly, AMPs from these marine biofilms may provide excellent therapeutic candidates for fighting antibiotic resistance, as evidenced by the fact that some of the bacteria employed for testing were clinically drug‐resistant. Most of these peptides show minimal toxicity to human cells, suggesting that they target specific components of bacterial cells. Furthermore, we provide evidence that the AMPs may target bacterial membranes, although certain unknown intracellular effects may also contribute to their bactericidal activity. Altogether, findings in the present study expand the repertoire of AMP molecules by focusing on marine biofilm bacteria, and the approach used here contributes to the acceleration of novel AMP discovery. However, limitations exist. Given that the candidate AMPs in this study were produced through chemical synthesis, the characteristics of naturally produced peptides may not have been fully replicated. For future direction, AMP modifications, such as cyclization and C‐terminal acetylation to improve their performance (e.g., resistance to protease digestion), will be conducted. It will be followed by animal experiments to further evaluate their potential to be novel drugs, as well as an in‐depth study of antimicrobial targets.

## CONCLUSION

This study successfully combines Ribo‐seq analysis with a new deep learning algorithm to predict AMPs from 713 pure cultured strains from marine biofilms, leading to the identification of 341 AMPs that show low sequence similarity with known AMPs. Synthesis and experimental validation confirm that 90% of these AMPs have antimicrobial activity. Mechanistic exploration reveals that the potent activity of the AMPs is likely afforded by disrupting the bacterial cell membrane. Therefore, culturable bacteria from marine biofilms stand for a trove of druggable AMPs.

## METHODS

### Marine biofilm sampling and bacterial isolation

#### Sampling

Samples of biofilms were collected from MP and ST surfaces along the coastal areas of Qingdao, China. Seven types of MP, including polymethyl methacrylate, polystyrene, polyethylene, polyethylene terephthalate, polypropylene, polyphenylene sulfide, and polycarbonate, having a diameter of 0.3 mm, were autoclaved for sterilizing and filled into nylon mesh bags. The bags were then immersed in coastal seawater to a depth of 1–2 m for 30 days. The biofilms attached to MP were transferred to the laboratory. Biofilms on ST surfaces immersed in the subtidal zone with a 1–2 m depth were also sampled. The biofilms were scraped off using sterile cotton tips, placed in sterile seawater, transferred immediately to the laboratory, and subjected to bacterial isolation.

#### Agar plate‐based strain isolation and identification

Bacterial cells collected from MP and ST biofilms were serially diluted (10, 10^2^, 10^3^, 10^4^, 10^5^, 10^6^, and 10^7^‐fold) before being plated on Marine Agar 2216E (BD Difco). After incubating the plates at 25°C in an environmental chamber (Jiangnan Instrument Factory), single colonies were chosen between the third and tenth day to be examined under a dissecting microscope (Motic China Group) for morphological characteristics. Selective colony types were taken and refined through a minimum of five subculturings on agar plates. After that, these colonies were moved to liquid Marine Broth 2216E (BD Difco) and given another 16 h to grow. Half of the cells were harvested for DNA extraction, while the other half preserved at −80°C DNA was extracted by heating the cells at 100°C for 5 min, and this was followed by PCR amplification of the 16S rRNA genes using the universal primers 27 F (5′‐AGAGTTTGATCCTGGCTCAG‐3′) and 1492 R (5′‐GGTTACCTTGTTACGACTT‐3′). The Sangon Biotech then carried out full‐length Sanger sequencing. Eventually, the single‐peak signaling 16S rRNA gene sequences that had been effectively constructed were kept for additional examination. The appropriate strains were kept at −80°C and were regarded as pure cultures.

#### Single‐cell strain isolation and identification

Single‐cell isolation was performed based on the limiting dilution method at Bioexploration Guangdong Limited. In the present case, flow cytometry determined cell density after bacterial cells from MP and ST biofilms were suspended in Marine Broth 2216E (BD Difco). The cells were then diluted to a concentration of around one cell per mL, and an automated dose dispenser (BYFY‐1DT8; Boyi Biotechnology Company) was used to dispense 0.4 mL of the diluted reagent into a 96‐well plate. This step ensured a “one‐cell‐one‐well” condition. The wells were finally sealed with a sterile sealing film and incubated at 25°C. Cell growth was observed every day by photographic monitoring. Between the 3rd and the 10th day, all opaque wells with an OD_600_ above 0.1 were labeled, and their contents were collected for DNA extraction and 16S rRNA gene amplification. The successfully assembled 16S rRNA gene sequences with single‐peak signals were eventually retained for further analysis. The corresponding strains were considered pure cultures and stored at −80°C.

#### Genome sequencing and compilation

CD‐HIT was used to identify duplicated sequences based on the 16S rRNA genes. The CD‐HIT software (version 4.8.1) [38] was utilized, and the results were validated using BLASTn. The QIIME2 scripts “multiple_rarefactions.py,” “alpha_diversity.py,” and “collate_alpha.py” [39] were used to generate OTU‐sequence rarefaction curves. The curves were produced at intervals of 100 with 10 repetitions. Whole genome sequencing was performed using DNA obtained from nonredundant strains. The TIANamp Genomic DNA Kit (Tiangen Biotech) was used to extract DNA from cultured strains. To extract DNA from bacterial strains with low DNA yield, 10 mg/mL of lysozyme was included before the DNA extraction process, following the stated instructions provided with the kit. The genome was sequenced using NovaSeq. 6000 equipment at Novogene Bioinformatics Institute in China. Each genome yielded 1–2 Gb of 150‐bp paired‐end reads. The sequences were subjected to quality control using the NGS QC Toolkit (version 2.0) [40], where the “IlluQC.pl” command was executed on paired‐end reads in FASTQ files with a Q20 cutoff. Then, high‐quality reads were assembled using SPAdes (version 3.0.0) [41] under a series of kmers, including 61, 71, 81, 91, and 101. The assembled genomes were assessed for completeness and potential contamination using CheckM2 (version 1.0.1) [42] with default parameters. Only genomes with >95% completeness and <5% contamination were used for further analysis. The genomes were subjected to taxonomy annotation using the “classify_wf” command in GTDB‐Tk (version 2.1.0) [43]. Redundant genomes were analyzed using the fastANI software (version 1.33), and those with ANI values > 99.9% were removed [44]. The plots of taxonomic affiliations were drawn using ImageGP [45]. The phylogenetic tree was generated based on 120 bacterial universal marker gene sets using GTDB‐Tk and visualized in iTOL [46]. For the analysis of a single genome, Prodigal (version 2.6.3) [49] was used to predict ORFs and retrieve both the ORFs and their matching protein sequences, specifically focusing on those that are closed‐ended. Functional gene annotation was performed by searching for the protein sequences against prokaryotes. The pep database was obtained from the KEGG FTP Academic Subscription in Japan [47], specifically the 2022 published version. It was documented using DIAMOND (version 0.9.14.115) [48], using an *E*‐value threshold of 1*e*−7.

### Ribo‐seq and sORF prediction

A total of 713 strains obtained from marine biofilms were cultured for one night in marine broth 2216E at a temperature of 25°C and a speed of 180 revolutions per minute in a shaker. After entering the logarithmic growth phase, the cell densities of the cultures were determined at 600 nm and then corrected to around 0.2 using the sterilized 2216E liquid media. The exact quantities of the strain cultures from the identical biofilm niche were combined. As a result, two combinations were acquired, one comprising strains from MP biofilms and the other from ST biofilms. The mixtures were seeded into fresh 2216E media in six‐well plates and grown under static conditions at 25°C for an additional 16 h, which allowed biofilm formation on the bottom of the wells. The two cultures were centrifuged and resuspended in 2216E media to collect the cells. Next, cycloheximide was added and incubated for 1 min, then centrifuged and resuspended in pre‐cooled phosphate‐buffered saline containing cycloheximide for another 1 min. After centrifugation, the supernatant was discarded, and the bacterial fluid was collected and subjected to quick freezing in nitrogen. Low‐concentration RNase treatment of the ribosome‐nascent peptide chain complex broke down RNA fragments lacking ribosome coverage. Using an Illumina NovaSeq platform at Novogene, small RNA fragments translocated and shielded by ribosomes have been identified under ribo‐seq. Finally, 25.20 and 27.32 Gb data sets were collected for the MP and ST biofilm‐derived strain combinations.

The CDS for the genomes of 378 ST and 335 MP strains were predicted using Prodigal with default parameters, merging them into two CDS libraries: ST and MP. These libraries were matched with the Ribo‐seq data obtained from ST and MP using bowtie2 (version 2.4.4) [50]. The relative abundance of each strain was determined by dividing the number of sequences that matched each strain by the total number of matched sequences. In addition, the number of sequences matched for different families in ST and MP was determined independently based on the phylogenetic classification of the strains.

sORFs were predicted from the 713 genomes using the getorf function of the EMBOSS (version 6.6.0.0) [51]. A range of amino acid sequences with lengths between 5 and 40 was allowed. The value of the “‐find” argument was set to 2, and the universal bacterial codon table was used. Duplicate sequences were removed using the rmdup function in Seqkit (version 2.1.1) [52]. The Ribo‐seq data sets were aligned onto the sORFs sequences by bowtie2. Sequences with matched bases to matched sequence length ratio of ≥2 were defined as expressed sORFs, while duplicated sORFs were removed using Seqkit.

### AMP sequence collection from public databases

#### AMP

AMP sequences were collected from four public databases: APD [13], DBAASP [53], dbAMP [54], and DRAMP [55], encompassing most AMP sequences from animals, plants, microbes, and fungi (until 2023.10.12). All collected sequences (*n* = 13,405) were compressed into one data file, from which sequences <5 amino acids or >40 amino acids, as well as those not containing antibacterial activity tags (e.g., only with antifungal or anticancer activity tags) were removed. Consequently, a total of 4025 nonredundant AMP sequences were obtained.

#### Non‐AMP

Peptides with non‐AMP activities were downloaded from the UniProt database (http://www.uniprot.org). After filtering sequences labeled as antibacterial, antibiotic, antiviral, antifungal, effector, or secretion, only sequences with ≤40 amino acids labeled with no antibacterial activity were retained. Repeated sequences were removed using CD‐HIT. Sequences identical to those in the AMP data set were removed, and a non‐AMP data file containing 40,291 sequences was generated.

#### Data set partitioning

The data set containing both the AMP and non‐AMP sequences was divided into three groups: training, validation, and testing. The ratio of samples in each group was 6:2:2. The training group and the validation group were used to optimize the performance of the model and choose the hyperparameters. The testing group was separate from the other two groups and used explicitly for conducting the final performance testing of the model.

### Model construction and evaluation

All peptide sequences in the AMP and non‐AMP files were converted into a computer‐readable format: (1) 20 different amino acids were converted into corresponding numbers; (2) at the end of sequences containing less than 40 amino acids, “0” was added to fix all the sequences to 40 amino acids; and (3) for the tags of AMPs, “1” was used, while for the tags of non‐AMPs, “0” was used.

Four neural network‐based models were built with TensorFlow (version 2.11.0) [56]. The initial layer of all models consisted of an embedding layer utilized to transform the sequence into an embedding vector. In the CNN model, the embedding layer was succeeded by a one‐dimensional convolution layer and a pooling layer to extract sequence information. Two fully connected layers were added using the relu activation function. The internal L2 regularization was adjusted to 0.02, and a sigmoid activation function was used to produce the prediction results. In the CNN‐BiLSTM model, two layers of BiLSTM were added to the CNN model, the L2 regularization was set to 0.02 within the BiLSTM unit, and two fully connected layers were then used for output. In the CNN‐Attention model, a dropout layer was added following the convolutional pooling layer based on the CNN model. Then, the Attention layer and completely connected layer were introduced. Finally, the CNN‐BiLSTM‐Attention model was developed by integrating CNN, BiLSTM, and Attention layers to create a multilayer structural model. The model consisted of a convolutional pooling layer, a dropout layer, two BiLSTM layers, an Attention layer, and three fully connected layers.

The loss function used for all models is binary cross‐entropy, and the ADAM optimizer is employed with default values, except for the learning rate set to 1e−4. The selection of hyperparameters is performed by a random search methodology. The number of neurons in the embedding layer is varied within the range of 64 to 128. The number of units, kernel size, and stride of the convolutional layer is changed within the ranges of 64–128, 2–8, and 1–8, respectively. The pooling layer's kernel size and stride are 2–8. The CNN model is configured with 20–100 units for the first feedforward neural network layer and 20–64 units for the second feedforward neural network layer. The CNN‐BiLSTM model is configured with a size of 64–128 and 20–64. The CNN‐BiLSTM‐Attention model has a configuration of 64–256 for the CNN layer and 20–64 for the BiLSTM layer. The dropout rate for the CNN‐Attention and CNN‐BiLSTM‐Attention models falls from 0.1 to 0.3. The CNN and CNN‐Attention models are trained for 100 epochs, whereas the others are trained for 30 epochs. We set the number of iterations for the random search to 200 and applied fivefold cross‐validation. All models were trained using a batch size of 64. An early stopping procedure was implemented with a patience value of 5 to minimize overfitting during training. All models demonstrate rapid convergence during the training process. The prediction probability threshold for all models was set to 0.5. The prediction probability threshold for all models was set to 0.5: values > 0.5 indicated AMPs, while ≤0.5 indicated non‐AMPs.

The performance of different models was tested by calculating the confusion matrix through TensorFlow. The accuracy, precision, recall, and MCC evaluation parameters were calculated employing scikit‐learn (version 1.2.2) [57]. These coefficients evaluated the model using the number of true positives (TP), true negatives (TN), false positives (FP), and false negatives (FN) in a confusion matrix.

$$\mathrm{Accuracy}=\frac{\mathrm{TP}+\mathrm{TN}}{\mathrm{TP}+\mathrm{FP}+\mathrm{TN}+\mathrm{FN}},$$

$$\mathrm{Precision}=\frac{\mathrm{TP}}{\mathrm{TP}+\mathrm{FP}},$$

$$\mathrm{Recall}=\frac{\mathrm{TP}}{\mathrm{TP}+\mathrm{TN}},$$

$$\mathrm{MCC}=\frac{\mathrm{TP}\times \mathrm{TN}-\mathrm{FP}\times \mathrm{FN}}{\sqrt{\left(\mathrm{TP}+\mathrm{FP})(\mathrm{TP}+\mathrm{FN})(\mathrm{TN}+\mathrm{FP})(\mathrm{TN}+\mathrm{FN}\right)}}.$$

In the scikit‐learn library, the Area Under the Precision‐Recall Curve (AUPRC) is used as an evaluation metric to demonstrate the balance between precision and recall at various classification levels. Furthermore, the AUPRC was applied to evaluate the model's effectiveness when handling data sets that exhibit imbalanced categories. Comparison with previously published models AMPActiPred [58], Amplify [59], Macrel [60], CAMP [61], AMP Scanner [9], AMPpred‐MFA [62], AMPfun [63], and Ampir [64] were conducted by calculating the test data set prepared in the present study.

### Candidate AMP prediction and sequence property analysis

Before assessing the prediction models, peptide sequences that Ribo‐seq revealed had their net charges determined. Amino acids that are positively charged (Lys, His, and Arg) have a charge of “1,” negatively charged (Glu and Asp) have a charge of “−1,” and neutral amino acids have a charge of “0.” Peptides with net charge >2 were used as queries for predicting AMPs using the CNN‐BiLSTM‐Attention model. Those with a probability score of >0.5 were considered candidate AMPs. The candidate AMPs were compared with those in the training data sets based on sequence similarity and amino acid frequency. Sequence similarity was assessed using the EMBOSS function with default parameters. Variations in sequence property, including global hydrophobicity and hydrophobic moment, charge, charge density, isoelectric point, instability index, aromaticity, aliphatic index, Boman index, and hydrophobic ratio, were also assessed using GlobalAnalysis and GlobalDescriptor in the modlamp package (version 4.3.0) [65].

### Peptide synthesis and antibacterial experiment

Sangon Biotech synthesized the potential AMPs using Fmoc solid‐phase synthesis techniques. Mass spectrometry and high‐performance liquid chromatography resolved AMP molecular weight and purity. Every peptide's purity exceeded 95%. Reconstituted in ultrapure water, the peptide powder obtained following lyophilization formed a 2 mg/mL solution.

Eight bacterial strains were utilized in the antibacterial experiment. *S. aureus* ATCC 12600, *B. subtilis* ATCC 23857, *E. coli* ATCC 11775, and *A. baumannii* ATCC 19606 were purchased from Shanghai Beinuo Biology. *M. luteus* ATCC 4698 and *S. bongori* ATCC43975 were purchased from Shanghai Beisi Biotechnology. *V. owensii* ems001 and *V. alginolyticus* xv22 from the intestines of diseased shrimps were isolated in our laboratory. All strains were cultured in the cation‐adjusted Mueller‐Hinton broth (CAMHB) at 37°C to the logarithmic growth phase (OD_600_ = 0.5). Then, bacteria were gradient‐diluted using fresh CAMHB to a bacterial density of 5 × 10^5^ CFU/mL. Antibacterial experiments were performed in a 96‐well plate with 200 μg/mL final peptide concentration. Four experimental groups were set up, including a blank control (200 μL CAMHB), a negative control (180 μL CAMHB plus 20 μL peptide), a positive control (180 μL bacterial culture plus 20 μL ultrapure water), and an experimental group (180 μL bacterial culture plus 20 μL peptide). Three biological replicates were conducted for each group, and OD_600_ values were measured after incubation at 37°C for 12 h. Every observed value was removed from the blank control group value. The value of the experimental group was then subtracted from the value of the negative control group therefore eradicating the effect of the peptide on the OD measurement. Student's *t*‐test allowed the estimation of a notable variation between the experimental and positive groups using a *p*‐value of 0.05 as the limit. MIC was assessed using a set of peptide concentrations ranging from 0.5 to 200 μg/mL. The MIC of a peptide against a bacterium was determined as the minimum peptide concentration at which there was no visible bacterial growth.

### Cytotoxicity assay

Human normal colon epithelial cells (NCM‐460) were purchased from the BeNa Culture Collection. Cytotoxicity was measured using the 3‐[4,5‐dimethylthiazol‐2‐yl]−2,5 diphenyl tetrazolium bromide (MTT) assay. The NCM‐460 cells were cultured in the Dulbecco's modified Eagle's medium (DMEM)‐H complete culture medium (90% DMEM‐H, 10% FBS, 10,000 U/mL penicillin, and 10,000 μg/mL streptomycin) to logarithmic growth phase. The suspension of cells was prepared at 10^5^ cells/100 μL/well in a 96‐well plate. The peptides selected for the assay were dissolved in the DMEM‐H medium to prepare a 25 mg/mL stock solution, diluted for multiple gradients of 4–512 μg/mL. Afterwards, the peptides that had been diluted (100 μL/well) were introduced into the cells residing on a 96‐well plate. The DMEM‐H medium served as the blank group, the cell suspension as the control group, and the cell suspension combined with the peptide as the experimental group. Each group was conducted in three separate replicates. After 48 h of culture, 10 μL MTT was added to each well and incubated further at 37°C for 4 h. Then 100 μL Formazan solution was added and incubated further at 37°C for 4 h until all the purple crystals were dissolved. The absorbances were measured at OD_570_. Cell viability was determined by calculating cell viability using the following formula.

$$\begin{array}{cll} \text{Cell viability} & = & \frac{{\mathrm{OD}}_{570}(\text{experimental})-{\mathrm{OD}}_{570}(\text{blank})}{{\mathrm{OD}}_{570}(\text{control})-{\mathrm{OD}}_{570}(\text{blank})}\times \;100\%. \end{array}$$

Then, the CC50 value of each AMP molecule was calculated using an online tool: https://www.aatbio.com/tools/ic50-calculator.

### Hemolysis assay

Freshly collected sheep blood was centrifuged at 3000 rpm for 10 min, and the supernatant was removed. The pellet was washed and resuspended with normal saline water to produce a 2% blood cell solution. Then, 400 μL of peptide solution was added to 100 μL of 2% blood cell solution to achieve final peptide concentrations of 8, 16, 32, 64, and 128 μg/mL. Normal saline water and Triton X‐100 normal saline solution (2%) were used as negative and positive controls, respectively. The samples were incubated in a water bath at 37°C for 1 h, followed by centrifugation at 3000 rpm for 10 min. Then, 200 μL of the supernatant was transferred to a 96‐well plate, and OD values were measured at a wavelength of 545 nm. The hemolysis rate of each sample was calculated using the following formula:

$$\text{Hemolysis ratio}=\frac{{\text{OD}}_{545}(\text{experimental})-{\text{OD}}_{545}(\text{negative control})}{{\text{OD}}_{545}(\text{positive control})-{\text{OD}}_{545}(\text{negative control})}\times \;100\%.$$

### AMP structure prediction

The structure of the AMP was predicted using ColabFold [66], with num_relax set to 1, template_mode set to pdb100, msa_mode set to mmseqs_uniref_env, and pair_mode set to unpaired_paired. Helical wheel projection of the peptides was calculated using the online software Heliquest (https://heliquest.ipmc.cnrs.fr/cgi-bin/ComputParams.py).

### CD spectrometer measurement

Peptide solutions were prepared using 30% TFE, maintaining a final concentration of 20 μM. CD measurements were carried out on a circular dichroism spectrometer (Jasco Circular Dichroism Spectrophotometer J‐1500). A quartz cell with a 1 mm path length and a wavelength range of 190–260 nm was used for the measurements. The scanning speed was 200 nm/min and the bandwidth was fixed at 1 nm. The Means‐Movement smoothing filter was used to process the spectral data, and Spectra Manager's Secondary Structure Estimation function was used to estimate the secondary structure.

### SEM and CLSM measurement

*S. aureus* ATCC 12600 was cultured overnight at 37°C in LB medium and diluted to 10^8^ CFU/mL. The cell suspension was treated with AMP at a final concentration of 5 × MIC at 37°C for 1 h, with untreated *S. aureus* ATCC 12600 as the control. The samples were then centrifuged at 4000 rpm for 5 min at room temperature, the supernatant was discarded, and the pellet was resuspended in phosphate‐buffered saline (PBS) (pH 7.0). Following two PBS washes of the samples, the cell pellet was again suspended in a 2.5% glutaraldehyde solution. The material was spread out on a glass slide and dried in two sessions using 100% ethanol and one treatment each of 30%, 50%, 70%, 80%, and 100% ethanol for 10 min. After that, isoamyl acetate was substituted for the samples. After critical point drying with CO_2_ and gold sputter coating, the samples were observed using SEM (Tescan Vega3).

*S. aureus* ATCC 12600 was cultured overnight in LB medium, and the OD was then adjusted to 0.6. The cells were treated with 1 × MIC of the peptide and incubated with shaking at 37°C for 40 min. Then, 1 μg/mL FM4‐64 was added, and the mixture was incubated on ice for 40 min. Subsequently, 20 μg/mL PI and 2 μg/mL DAPI were added, and the mixture was incubated on ice for an additional 15 min. The cells were then collected by centrifugation at 3000 rpm for 6 min and resuspended in LB medium. The cell suspension was air‐dried on a glass slide and mounted with an anti‐fade mounting medium. The samples were observed using a CLSM (Zeiss LSM980) at the Fang Zongxi Center, Ocean University of China.

## STATISTIC ANALYSIS

Statistical analyses were conducted using python software (https://www.python.org/), version 3.9.16. Visualizations were primarily created with the “matplotlib” package in python. In model building, the “TensorFlow” package (version 2.11.0) in Python was used for construction and parameter optimization. The “sklearn.metrics” package (version 1.2.2) was applied to evaluate the model's predictive performance using precision, accuracy, recall, the Matthews Correlation Coefficient, and the precision‐recall curve. Global hydrophobicity and the global hydrophobic moment were visualized using the “modlamp.analysis.GlobalAnalysis” module (version 4.3.0), while other sequence properties were calculated and visualized with the “modlamp.descriptors.GlobalDescriptor” module (version 4.3.0). To assess differences between groups, an independent‐samples equal variance two‐tailed *t*‐test was used for data with multiple parallel groups. A *p* < 0.05 indicates statistical significance, *p* < 0.01 represents highly significant differences, and *p* < 0.001 indicates extremely significant differences.

## AUTHOR CONTRIBUTIONS

**Shen Fan**: Data curation; writing—original draft; methodology. **Peng Qin**: Data curation. **Jie Lu**: Data curation. **Shuaitao Wang**: Data curation. **Jie Zhang**: Data curation. **Yan Wang**: Conceptualization. **Aifang Cheng**: Conceptualization. **Yan Cao**: Conceptualization. **Wei Ding**: Resources; supervision. **Weipeng Zhang**: Supervision; writing—review and editing; resources.

## CONFLICT OF INTEREST STATEMENT

The authors declare no conflict of interest.

## ETHICS STATEMENT

No animals or humans were involved in this study.

## Supporting information


**Figure S1:** Bacterial colonies formed on an agar plate after dilution.
**Figure S2:** Scanning electron microscope observation on three selected strains.
**Figure S3:** Rarefaction analysis between the number of 16S rRNA gene sequences of the isolated strains and the respective operational taxonomic units (OTUs).
**Figure S4:** Quality information of the 713 high‐quality genomes of bacteria isolated from microplastic (MP) and stone (ST) biofilms.
**Figure S5:** Pairwise average nucleotide identity (ANI) analysis.
**Figure S6:** Classification of the marine biofilm bacterial genome at the family level.
**Figure S7:** Classification of the marine biofilm bacterial genome at the genus level.
**Figure S8:** Venn analysis of family‐ and genus‐level affiliations between the MP‐ and ST‐derived bacterial genomes.
**Figure S9:** Protein‐coding gene prediction and annotation.
**Figure S10:** Data source of the AMPs from four databases used for training the deep learning model and their taxonomic affiliations.
**Figure S11:** Structure of the four deep learning models constructed in the present study.
**Figure S12:** Performance comparison between our model (the CNN‐BiLSTM‐Attention model) and previously reported AMP prediction models.
**Figure S13:** Recruitment of Ribo‐seq reads by genomes of the 713 bacterial isolates.
**Figure S14:** Family affiliation of the expressed small open reading frames (sORFs) encode peptides with net charges >2.
**Figure S15:** Family‐level affiliation of the sORFs identified as candidate AMPs.
**Figure S16:** Sequence identities between the MP‐ and ST‐derived candidate AMPs.
**Figure S17:** Comparative view of the amino acid frequency between the MP‐ (blue) and ST‐derived (orange) candidate AMPs.
**Figure S18:** Comparative view of length, molecular weight, charge, Instability index, aromaticity, Boman index, charge density, isoelectric point, aliphatic index, and hydrophobic ratio between the MP‐ (blue) and ST‐derived (orange) candidate AMPs.
**Figure S19:** Secondary structures of the candidate AMPs predicted by AlphaFold2.
**Figure S20:** Helical wheel projection of the AMP calculated by HeliQuest.

**Table S1:** Genomic information of the marine biofilm‐derived pure cultures.
**Table S2:** Information of the Ribo‐seq data.
**Table S3:** Taxonomic source and sequence of the 341 candidate AMPs.
**Table S4:** Information of the eight pathogenic strains used for antimicrobial experiments.

## Data Availability

The strains reported in this paper have been preserved in China General Microbiological Culture Collection Center (CGMCC, https://cgmcc.net/). All the strains can be freely accessed through the strain names listed in Table S1. The genome sequence data have been deposited in the Genome Warehouse (GWH) in National Genomics Data Center (NGDC), Beijing Institute of Genomics (BIG), Chinese Academy of Sciences (CAS), under the accession number PRJCA019328 (https://ngdc.cncb.ac.cn/gwh/search/advanced/result?search_category=&search_term=&source=0&query_box=PRJCA019328). Please see Table S1 for the accession number for each genome. Alternatively, the genomes can be downloaded from figshare with one click (https://figshare.com/s/decb10d44c2e8657cadf?file=49018204). The deep learning model has been deposited in github (https://github.com/FAFFASS/AMP_prediction). Supplementary materials (figures, tables, graphical abstract, slides, videos, Chinese translated version, and update materials) may be found in the online DOI or iMeta Science http://www.imeta.science/. The data that support the findings of this study are openly available in Genome Warehouse in National Genomics Data Center at https://ngdc.cncb.ac.cn/gwh/search/advanced/result?search_category=&search_term=&source=0&query_box=PRJCA019328, reference number PRJCA019328.
