# Supplementary material for: Bioprospecting of culturable marine biofilm bacteria for novel antimicrobial peptides
Source: Imeta. 2024 Oct 17;3(6):e244. doi: 10.1002/imt2.244 (PMC11683478; doi:10.1002/imt2.244)
Supplement: Supplementary file 1 — Figure S1: Bacterial colonies formed on an agar plate after dilution. Figure S2: Scanning electron microscope observation on three selected strains. Figure S3: Rarefaction analysis between the number of 16S rRNA gene sequences of the isolated strains and the respective operational taxonomic units (OTUs). Figure S4: Quality information of the 713 high‐quality genomes of bacteria isolated from microplastic (MP) and stone (ST) biofilms. Figure S5: Pairwise average nucleotide identity (ANI) analysis. Figure S6: Classification of the marine biofilm bacterial genome at the family level. Figure S7: Classification of the marine biofilm bacterial genome at the genus level. Figure S8: Venn analysis of family‐ and genus‐level affiliations between the MP‐ and ST‐derived bacterial genomes. Figure S9: Protein‐coding gene prediction and annotation. Figure S10: Data source of the AMPs from four databases used for training the deep learning model and their taxonomic affiliations. Figure S11: Structure of the four deep learning models constructed in the present study. Figure S12: Performance comparison between our model (the CNN‐BiLSTM‐Attention model) and previously reported AMP prediction models. Figure S13: Recruitment of Ribo‐seq reads by genomes of the 713 bacterial isolates. Figure S14: Family affiliation of the expressed small open reading frames (sORFs) encode peptides with net charges >2. Figure S15: Family‐level affiliation of the sORFs identified as candidate AMPs. Figure S16: Sequence identities between the MP‐ and ST‐derived candidate AMPs. Figure S17: Comparative view of the amino acid frequency between the MP‐ (blue) and ST‐derived (orange) candidate AMPs. Figure S18: Comparative view of length, molecular weight, charge, Instability index, aromaticity, Boman index, charge density, isoelectric point, aliphatic index, and hydrophobic ratio between the MP‐ (blue) and ST‐derived (orange) candidate AMPs. Figure S19: Secondary structures of the candidate AMPs predicted by Al [file IMT2-3-e244-s001.docx]

Supporting information to：

**Bioprospecting of culturable marine biofilm bacteria for novel antimicrobial peptides**

Running title: Antimicrobial peptide from marine biofilm

Shen Fan^1,#^, Peng Qin^1,#^, Jie Lu^1,#^, Shuaitao Wang^1,#^, Jie Zhang^1,#^, Yan Wang^1^, Aifang Cheng^3^, Yan Cao^4^, Wei Ding^2,*^, Weipeng Zhang^1,*^

^1^MOE Key Laboratory of Evolution & Marine Biodiversity and Institute of Evolution & Marine Biodiversity, Ocean University of China, Qingdao, 266000 China

^2^MOE Key Laboratory of Marine Genetics & Breeding and College of Marine Life Sciences, Ocean University of China, Qingdao, 266000 China

^3^Department of Biomedical Sciences, Faculty of Health Sciences, University of Macau, Taipa, Macao SAR, 999078 China

^4^College of Pulmonary & Critical Care Medicine, Chinese PLA General Hospital, Beijing, 100091 China

^#^These authors contributed equally: Shen Fan, Peng Qin, Jie Lu, Shuaitao Wang, Jie Zhang

*Correspondence: [zhangweipeng@ouc.edu.cn](mailto:zhangweipeng@ouc.edu.cn) (Weipeng Zhang), [dingwei@ouc.edu.cn](mailto:dingwei@ouc.edu.cn) (Wei Ding)

**Figure S1** Bacterial colonies formed on an agar plate after dilution. This is from a biofilm community that was collected from the surface of a subtidal stone. Blue boxes indicate colonies overlaid with others.

**Figure S2** Scanning electron microscope observation on three selected strains. The phenotypes of strains from three distinct genera were observed.

**Figure S3** Rarefaction analysis between the number of 16S rRNA gene sequences of the isolated strains and the respective operational taxonomic units (OTUs). The OTUs were clustered at a 97% similarity level.

**Figure S4** Quality information of the 713 high-quality genomes of bacteria isolated from microplastic (MP) and stone (ST) biofilms. Genome size (A), number of contigs (B), maximum size of the contig (C), and the N50 of contigs (D) are given. In a boxplot, the central line represents the median, bounds represent the upper and lower quartiles, whiskers represent the maximum and minimum, and the cross indicates the average.

**Figure S5** Pairwise average nucleotide identity (ANI) analysis. Each pair of the 713 genomes was compared using fastANI to produce 253,828 comparisons. (A) ANI values are shown in a heatmap. (B) ANI contributions are shown in bar charts.

**Figure S6** Classification of the marine biofilm bacterial genome at the family level. The 713 genomes, including 335 from microplastics and 378 from stone biofilms, were classified into 44 families.

**Figure S7** Classification of the marine biofilm bacterial genome at the genus level. The 713 genomes were classified into 134 genera.

**Figure S8** Venn analysis of family- and genus-level affiliations between the MP- and ST-derived bacterial genomes. The 713 genomes were classified into 44 families and 134 genera.

**Figure S9** Protein-coding gene prediction and annotation. (A) Number of open reading frames (ORFs). (B) Number of annotated Kyoto Encyclopedia of Genes and Genomes (KEGGs). (C) The ratio of KEGG-annotated ORFs to all ORFs. In a boxplot, the central line represents the median, bounds represent the upper and lower quartiles, whiskers represent the maximum and minimum, and the cross indicates the average.

**Figure S10** Data source of the AMPs from four databases used for training the deep learning model and their taxonomic affiliations. In total, 4025 sequences were downloaded from the four databases.

**Figure S11** Structure the four deep learning models constructed in the present study. (A) A one-dimensional CNN model. (B) Attention models were introduced to capture key sequence features. (C) The one-dimensional CNN model was augmented with two BiLSTM layers that simultaneously consider the peptide sequences' time dependence and global context information. (D) A hybrid model incorporating CNN, BiLSTM, and Attention layers.

**Figure S12** Performance comparison between our model (the CNN-BiLSTM-Attention model) and previously reported AMP prediction models. The performances were compared based on accuracy (A), precision (B), recall (C), and matthews correlation coefficient (D) when calculating the same dataset.

**Figure S13** Recruitment of Ribo-seq reads by genomes of the 713 bacterial isolates. The Ribo-seq data were aligned to the genomic CDS using Bowtie2. The sequence match counts and the respective accumulation curve are displayed.

**Figure S14** Family affiliation of the expressed small open reading frames (sORFs) encode peptides with net charges > 2. Using Ribo-seq and charge screening, 80,430 sORFs distributed among 44 families were identified. The top 12 families accounted for 90% of sequences.

**Figure S15** Family-level affiliation of the sORFs identified as candidate AMPs. In total, 341 candidate AMPs across 33 families were determined using the CNN-BiLSTM-Attention model.

**Figure S16** Sequence identities between the MP- and ST-derived candidate AMPs. The sequence identities were determined using BLASTp.

**Figure S17** Comparative view of the amino acid frequency between the MP- (blue) and ST-derived (orange) candidate AMPs. Similar amino acid frequency was observed between AMPs from the two biofilm niches.

**Figure S18** Comparative view of sequence features of the MP- (blue) and ST-derived (orange) candidate AMPs. Length, molecular weight, charge, instability index, aromaticity, Boman index, charge density, isoelectric point, aliphatic index, and hydrophobic ratio were calculated.

**Figure S19** Secondary structures of the candidate AMPs predicted by AlphaFold2. Four candidate AMPs with potent antimicrobial activities were analyzed.

**Figure S20** Helical wheel projection of the AMP calculated by HeliQuest. Yellow dots represent hydrophobic residues, blue and purple dots represent polar or charged residues, and green dots indicate amino acids that are potentially important for structural stability. "N" and "C" represent the N- and C-terminus.
